# Horned Woman

**Published:** 1853-07

**Authors:** 


### *Horned Woman.*

Dr. Austin L. Sands, of Cold Spring, New York, gives a case of "Ichthyosis cornea," which was of sixteen years growth.

"On inspection, it was found to arise from, or rather immediately over, the occipital protuberance, and to extend downwards and backwards about four inches, and then curling upon itself, terminating in a rough sulcated extremity. On handling it, it was found to be very hard, solid, and, when struck with the handle of a scalpel, gave a sharp clear sound; its attachments to the occiput did not appear to be very firm, as they allowed slight motion. Seeing no difficulty in the way of its immediate removal, an eliptical incision was made on each side of it through the integuments down to the periosteum, and then slipping the scalpel between the base of the horn and the bone, it was easily removed. There was little hemorrhage, and the wound healed kindly by first intention.

On examination after removal, the horn was found to measure *six and three quarter* inches in length, and *three* inches in circumference at the base."

